# Obesity and chronic kidney disease: A current review

**DOI:** 10.1002/osp4.629

**Published:** 2022-07-19

**Authors:** Saira Nawaz, Rajkumar Chinnadurai, Saif Al‐Chalabi, Philip Evans, Philip A. Kalra, Akheel A. Syed, Smeeta Sinha

**Affiliations:** ^1^ Faculty of Biology, Medicine and Health University of Manchester Manchester UK; ^2^ Department of Renal Medicine Salford Royal Hospital Northern Care Alliance NHS Foundation Trust Salford UK; ^3^ Department of Renal Medicine Liverpool University Hospitals NHS Foundation Trust Liverpool UK; ^4^ Department of Diabetes, Endocrinology and Obesity Medicine Salford Royal Hospital Northern Care Alliance NHS Foundation Trust Salford UK

**Keywords:** chronic kidney disease, dialysis, obesity, transplantation

## Abstract

**Background:**

Obesity poses significant challenges to healthcare globally, particularly through its bi‐directional relationship with co‐morbid metabolic conditions such as type 2 diabetes and hypertension. There is also emerging evidence of an association between obesity and chronic kidney disease (CKD) which is less well characterized.

**Methods:**

A literature search of electronic libraries was conducted to identify and present a narrative review of the interplay between obesity and CKD.

**Findings:**

Obesity may predispose to CKD directly as it is linked to the histopathological finding of obesity‐related glomerulopathy and indirectly through its widely recognized complications such as atherosclerosis, hypertension, and type 2 diabetes. The biochemical and endocrine products of adipose tissue contribute to pathophysiological processes such as inflammation, oxidative stress, endothelial dysfunction, and proteinuria. The prevention and management of obesity may prove critical in counteracting both the development and advancement of CKD. Moreover, measures of abdominal adiposity such as waist circumference, are generally associated with worse morbidity and mortality in individuals receiving maintenance hemodialysis.

**Conclusion:**

Obesity is a risk factor for the onset and progression of CKD and should be recognized as a potential target for a preventative public health approach to reduce CKD rates within the general population. Future research should focus on the use of glucagon‐like peptide‐1 receptor agonists and sodium–glucose cotransporter 2 inhibitors in patients with CKD and obesity due to their multi‐faceted actions on major outcomes.

## Visual Abstract

Obesity poses significant challenges to healthcare globally, particularly through its bi‐directional relationship with co‐morbid metabolic conditions such as type 2 diabetes and hypertension. In addition, it increases the risk of onset and progression of CKD through direct and indirect pathways. Therefore, obesity should be recognized as a potential target for a preventative public health approach to reduce CKD rates within the general population.

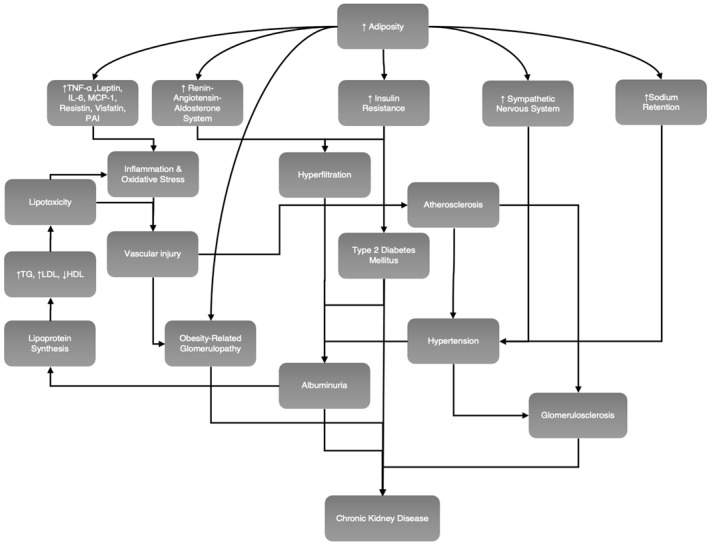

## 1 INTRODUCTION

Obesity is an important risk factor for premature death and the development of a plethora of non‐communicable diseases such as type 2 diabetes mellitus (T2DM), heart disease, hypertension, stroke, malignancy, and chronic kidney disease (CKD).^1^, ^2^ CKD is a global health issue associated with poor health outcomes and quality of life, as well as serious systemic complications including cardiovascular disease (CVD), metabolic bone disorder, anemia, and acid‐base and fluid imbalance.^3^, ^4^ It is a complex disease, delineated by the presence of abnormalities of kidney function or structure present for more than 3 months.^5^ The progressive and irreversible nature of CKD and end‐stage kidney disease (ESKD) mean that they are associated with considerable levels of morbidity, mortality and health resource expenditure^6^; therefore, avoidable risk factors for onset and progression of CKD and ESKD should be explored. As obesity is preventable,^7^, ^8^ patient education and communication with regards to good lifestyle management promotion, through nutritional intake and exercise, may play a substantial role in reducing the risk of onset of both CKD and obesity.

The rising prevalence of obesity has considerable implications for CKD.^9^ In England, during 2018–19, the estimated prevalence of CKD stages 3–5 in adults was 4.1%, whilst in Scotland it was 3.1%. Moreover, the incidence of patients receiving renal replacement therapy (RRT) of any form has increased over time throughout the United Kingdom.^10^ A longitudinal screening study in the United Kingdom primary care setting observed that greater than 40% of people living with CKD were undiagnosed without screening, arguing the need for more robust surveillance to promptly diagnose the disease which is frequently asymptomatic in its early stages.^11^ CKD is also a global concern and, in 2017, the results from a systematic review and meta‐analysis estimated the population of individuals with CKD across the planet to be 697.5 million. Meanwhile in the same year, estimated mortality from CKD was 1.2 million, constituting it as the 12th largest cause for deaths worldwide—an appreciable rise from the late twentieth century.^12^

## 2 ETIOLOGICAL FACTORS IN OBESITY

An excessive accumulation of body fat leads to a non‐optimal health state due to a greater risk of attaining diseases, target‐organ damage^1^, ^13^ and a shortened life expectancy. Data from the United States showed marked race and sex differences in observed years of life lost (YLL). White men aged 20–30 years with severe obesity had a maximum of 13 YLL compared to 8 YLL for White women of the same age. However, for Black individuals, men have significantly higher YLL at 20 and women have 5 YLL.^14^ In a study from Australia, higher body mass index (BMI) was associated with shorter life expectancy at any age for both men and women. The highest risk reported was for individuals in 20–39‐year age groups who had 8–10 YLL.^15^ Obesity predominates in the pathogenesis of metabolic abnormalities such as hyperglycaemia, insulin resistance, dyslipidaemia and atherosclerosis.^16^, ^17^

Overweight and obesity manifest more commonly in individuals with a *mono‐*, *oligo‐* or *polygenic* predisposition, and there are a number of genes implicated in the development of a heritable obesogenic phenotype.^18^ There is no accurate estimate of the scale of the genetic contribution to heritable obesity. However, a metanalysis of 25 family studies showed a BMI heritability estimate to range from 24% to 81%.^19^ Non‐syndromic, monogenic causes of obesity, though rare, are linked strongly to homozygous mutations in genes contributing to the formation of the brain‐adipose axis. These include loss of function mutations in genes which encode for components along the leptin‐melanocortin pathway such as *leptin (LEP)*, *leptin receptor (LEPR)*, *melanocortin 4 receptor (MC4R)*, *proconvertase 1 (PCSK1)* and proopiomelanocortin (*POMC*), resulting in severe, early‐onset and penetrant obesity.^20^, ^21^, ^22^, ^23^ Leptin is central to alerting the brain with regards to energy intake; it is a satiety‐inducing polypeptide produced mainly by adipocytes, and functions in the regulation of food intake, energy homeostasis and body mass.^21^ Oligogenic variants of obesity develop through defects in these genes when inherited in a heterozygous manner, and are characterized by greater variability in the spectrum of obesity along with more degree of dependence upon environmental factors.^22^ Oligogenic obesity accounts for approximately 3% of cases of obesity in adults and children.^23^

The majority of potentially heritable obesity however, is polygenic and this underpins most cases of “common” obesity.^23^ Here, it is the cumulative contribution of multiple loci in synergy with epigenetic modulation and importantly, a fat‐promoting lifestyle and environment, which bring about increased adipose deposition and raised BMI.^22^ For instance, the FTO gene was the first to be identified by genome‐wide association studies (GWAS) as a known and prominent contributor to polygenic obesity at a population level. SNPs on the first intron of this gene, mapped on chromosome 16, show a significant association with the onset of T2DM and increased BMI.^24^, ^25^ The use of GWAS technology has enabled greater pathophysiological understanding of polygenic obesity by delineating hundreds of novel polymorphisms which strongly influence BMI, although the current identities may explain a fraction of the total variance.^26^, ^27^ Similarly, certain genes which are implicated in monogenic obesity also display variations which influence BMI and contribute to the risk of attaining polygenic obesity.^28^, ^29^, ^30^, ^31^

A heritable link to obesity is supported strongly across the literature,^22^, ^23^ although the effects of environment and lifestyle factors are also crucial clinically. Increased adiposity is produced by a homeostatic imbalance between calorific input and energy expenditure resulting in weight gain due to a positive net energy balance. The prevalence of an obesogenic environment and overnutrition; coupled with lifestyle behaviors are implicated in the recent escalation of obesity globally.^32^, ^33^ Current United Kingdom guidelines recommend that the saturated fat content in the diet should be no greater than 11% of daily calorific input, whilst free sugars should form no more than 5%.^34^ Moreover, lack of physical activity, a rise of which has developed over the past decades, has shown significant correlation with raised BMI, CVD and metabolic syndrome in studies.^35^, ^36^ Certain polymorphisms of the FTO gene implicated in polygenic obesity are accentuated on an environmental background of physical inactivity and overnutrition, highlighting the interaction between genetics and environment in the development of obesity.^37^, ^38^

## 3 OBESITY AND KIDNEY DISEASE: RENAL HISTOPATHOLOGICAL CHANGES AND ETIOLOGICAL FACTORS

**FIGURE 1:**
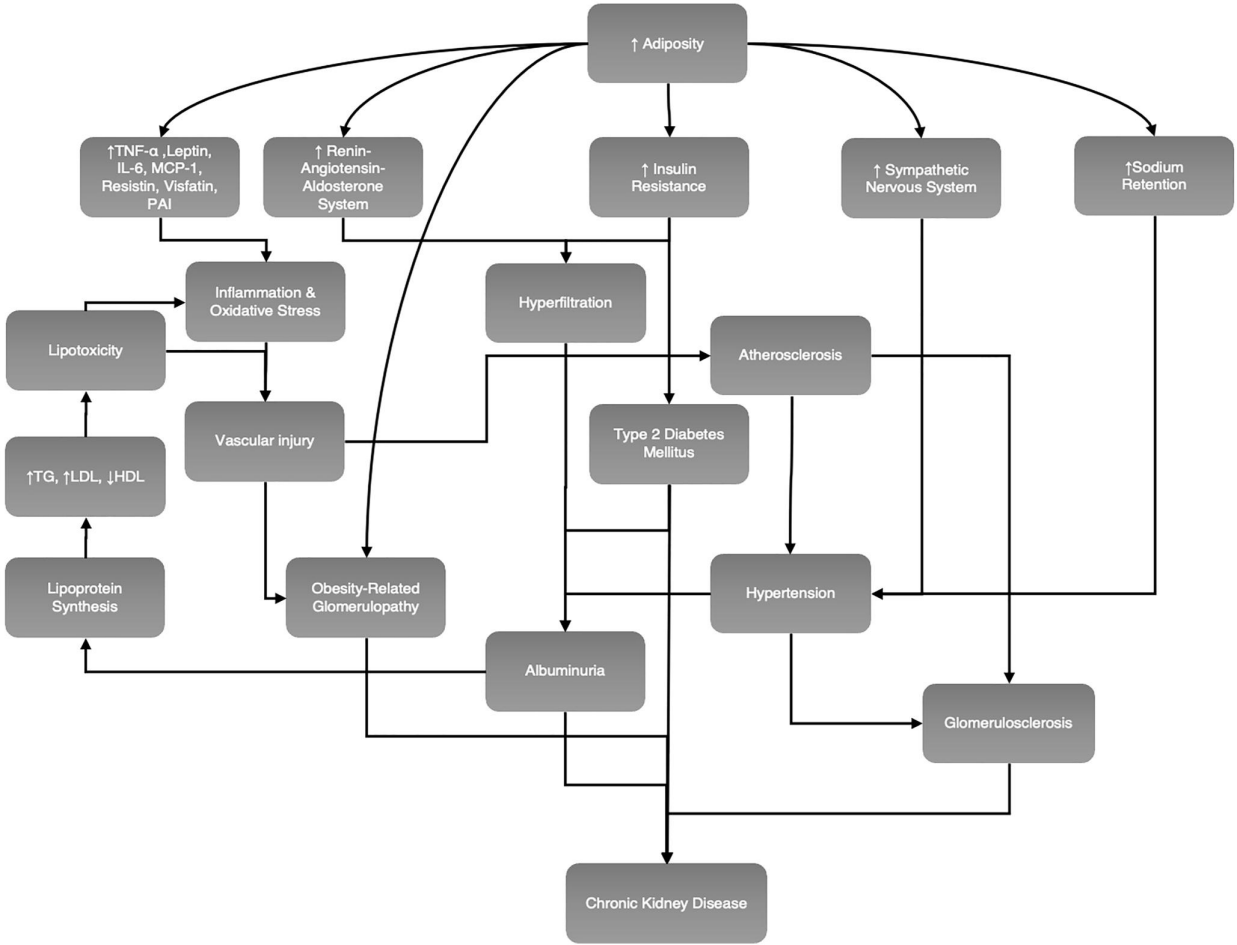
Direct and indirect mechanisms by which adiposity can perturb renal function and lead to kidney disease. HDL, high‐density lipoprotein; IL‐6, interleukin‐6; LDL, low‐density lipoprotein; MCP‐1, monocyte chemo‐attractant protein 1; PAI, plasminogen‐activator inhibitor; TG, triglycerides; TNF‐α, tumor necrosis factor alpha

Obesity impairs kidney function via the direct effects which adiposity exerts on the kidney, and indirectly due to systemic complications of obesity including diabetes mellitus, atherosclerosis and hypertension, Figure 1.^39^, ^40^, ^41^ Obesity can directly injure glomeruli via hemodynamic alterations primarily due to vasodilatation of the afferent arteriole and an increase in proximal tubular salt reabsorption. These changes cause glomerular hyperfiltration and ultimately proteinuria.^42^ The accelerating prevalence of obesity globally is paralleled by the increasing incidence of obesity‐related glomerulopathy (ORG); a diagnosis made upon the exclusion of clinical or histopathological evidence of other renal pathology,^43^ in individuals with a BMI greater than 30. ORG is characterized by glomerulomegaly presenting alone or with secondary focal segmental glomerulosclerosis found frequently in the glomerular peri‐hilar area.^44^ Biopsy studies have shown structural variations consistent with ORG, including glomerulomegaly, decreased podocyte density, and increased width of podocyte foot processes,^44^, ^45^ enlarged glomerular volume with a correspondingly marked decline in podocyte density,^45^, ^46^ increased mesangial matrix,^47^ and mesangial sclerosis and glomerular basement membrane thickening.^48^ The clinical features which occur secondary to these abnormalities include a gradual progression of sub‐nephrotic range proteinuria (<3.5 g/day) found alone or alongside wider renal insufficiency,^44^ though the presence of overt nephrotic syndrome in the context of ORG is rare.^42^

Underlying mechanisms leading to ORG are thought to include a greater renal hemodynamic and metabolic demand in obesity which causes hyperfiltration, increased proximal tubular sodium reabsorption, greater glomerular tuft volume and later, glomerulosclerosis.^42^, ^48^, ^49^ Indeed, hyperfiltration is understood to be a noteworthy contributor in the mechanism of renal damage in patients with obesity and has been observed independent of hypertension in humans.^50^ Obesity can cause increase in filtration fraction (FF) that results in hemoconcentration in the postglomerular circulation and increased oncotic pressure. In a human study on selected lean patients and those with obesity, the group of patients with obesity demonstrated higher glomerular filtration rate (GFR), renal plasma flow, and FF compared to the lean group. The study showed that the increased glomerular filtration led to increased postglomerular oncotic pressure and enhancement of proximal tubular sodium reabsorption.^50^ Physiological renal reaction to overcome this included compensatory renal vasodilation and increase in GFR. These processes will ultimately result in hypertension, albuminuria and more kidney damage.^50^, ^51^ Other processes understood to contribute to the observed glomerular hypertension and hyperfiltration within this context include insulin resistance, sympathetic nervous system over‐activity, and upregulation of the renin‐angiotensin system; this results in an elevated FF and unfavorable renal hemodynamics.^51^, ^52^, ^53^ As such, therapeutic interventions including blockade of the renin‐angiotensin system and sodium restriction can generally have a positive influence on renal hemodynamic status.^53^, ^54^

The absolute risk of individuals, to develop ORG is comparatively low; indicating that an underlying renal vulnerability increases susceptibility in certain individuals.^55^ Predisposing factors which increase the risk of developing ORG are generally understood to involve conditions which lower innate nephron numbers such as intrauterine growth restriction and low birth weight, impaired renal development and congenital renal abnormalities.^46^ These determinants, when paired with a period of disproportionate “catch‐up” growth after infancy will cause disparity between body mass to nephron ratio, increasing the risk of glomerular hyperfiltration and hypertension throughout later life in the setting of obesity.^56^, ^57^ Whilst epidemiological and observational studies have reported 4%–10% of patients with obesity have proteinuria, including microalbuminuria,^42^ the true incidence of ORG is unknown as guidelines and indications for renal biopsies vary globally. Consequently, the prevalence of ORG may be underestimated.

Obesity imposes a state of maladaptive persistent low‐grade inflammation, oxidative stress and cellular damage to peripheral tissues including the kidneys.^58^, ^59^ White adipose tissue is a complex and extremely functional endocrine organ derived from a host of cells comprising of adipocytes, endothelial cells, preadipocytes, leukocytes, macrophages, monocytes and fibroblasts.^60^ Inflammatory processes are mediated by the endogenous production of nephrotoxic adipose derived cytokines and mediators, namely tumor necrosis factor, leptin,^61^ interleukin 6 (IL‐6), monocyte chemo‐attractant protein 1, resistin, visfatin and plasminogen‐activator inhibitor, which cause renal injury,^41^, ^62^, ^63^, ^64^ Table 1.

**TABLE 1:** Potential mechanisms to explain the effects of adipocytokines, which are cell signaling molecules secreted by adipose tissue, on renal function

Adipocytokine	Relevance in kidney disease
Leptin	Hyperleptinemia is observed in patients with CKD and those undergoing dialysis; causing inflammation, glomerulosclerosis, proteinuria, increased sympathetic nervous activity and ROS stimulation.^61^, ^65^ Elevated serum leptin causes upregulation of type IV collagen and, TGF‐β1 in glomerular endothelial cells, causing these cells to proliferate.^66^ In glomerular mesangial cells, hyperleptinemia causes upregulation of the TGF‐β type II receptor, type I collagen synthesis, glucose uptake and mesangial cell hypertrophy. Both endothelial and mesangial cells increase release of extracellular matrix constituents in response to leptin.^67^
Resistin	Serum levels of resistin elevate across mild to advanced CKD and negatively associate with GFR, albumin and hematocrit.^68^, ^69^ Hyper‐resistinaemia increases release of pro‐inflammatory cytokines including C reactive protein, an acute phase reactant linked to endothelial cell dysfunction, coronary artery disease, adiposity and insulin resistance. A marked increase in TNF, lipoprotein‐associated phospholipase A2 and IL‐6 is also observed in response to raised serum resistin, conferring that resistin may be associated with sub‐clinical inflammation recognized in CKD.^68^, ^70^ Expression of resistin propagates adhesion molecules in vascular endothelial cells such as anti‐VCAM‐1 and anti‐ICAM‐1, and long pentraxin 3, the latter is also a marker of inflammation. Resistin's induction of VCAM‐1 and ICAM‐1 is directly inhibited by adiponectin. The extent to which this will affect renal function is unclear.^71^ Serum resistin has also found to correlate with variability in erythropoietin responsiveness in CKD patients.^72^
Visfatin	Visfatin, an adipocyte derived polypeptide hormone, is expressed preferentially in visceral fat.^73^, ^74^ Serum visfatin is associated with increased endothelial dysfunction seen in all stages of CKD, which is independent of insulin resistance and inflammation. It associates independently with VCAM‐1; a marker of damage to endothelia.^75^ In patients with CKD, higher levels of visfatin are associated with lower GFR, and increased triglyceride and low‐density lipoprotein; it is also proatherogenic in nature.^76^
TNF	TNF is a pro‐inflammatory, proatherogenic compound, released by macrophage cells; it correlates positively with CKD severity and associates with higher cystatin C and UACR.^77^, ^78^, ^79^, ^80^ The effects of TNF involve endothelial cell adhesion, ROS activation, increased albumin permeability, cytotoxicity, apoptosis and necrosis.^80^ Plays an active role in glomerular inflammation and fibrosis.^79^
IL‐6	IL‐6 is linked to greater CKD status, higher cystatin‐C, UACR and is implicated in both acute and long‐term inflammation. It is also associated with greater risk of incident CKD and raised in patients with kidney disease.^77^, ^81^ High levels of IL‐6 contribute to raised serum levels of fibroblast growth factor 23, which is found raised early in CKD and is associated with greater morbidity and mortality, and is linked to inflammatory processes.^82^, ^83^
MCP‐1	MCP‐1 is expressed in great abundance by visceral fat and implicated in CVD; MCP‐1 is released by macrophages and endothelial cells and recruits pro‐inflammatory cells.^84^ Plasma levels of MCP‐1 are observed to be raised in CKD and negatively associate with GFR and is independent as a risk factor for death in CKD patients.^85^
PAI	PAI is increased in CKD, and the glycoprotein is found to contribute to the promotion of renal fibrosis.^86^ PAI facilitates cell migration of inflammatory compounds such as monocytes and myofibroblasts within the renal interstitium, promoting fibrogenesis. It also induces fibrosis by inhibiting intravascular and tissue fibrinolytic processes.^87^ Both PAI‐1 and messenger ribonucleic acid protein are raised in chronic glomerulonephritis, diabetic nephropathy and FSGS. PAI‐1 abundance also correlates with degree of proteinuria.^88^
Adiponectin	Hypoadiponectinemia is observed in increasing visceral obesity and type 2 diabetes mellitus.^60^ Adiponectin is insulin sensitizing, anti‐inflammatory, antifibrogenic, anti‐atherogenic and cardioprotective.^89^ Circulating adiponectin is raised in CKD, perhaps due to factors such as reduced clearance which is evidenced by normalized levels post kidney transplantation.^60^ Adiponectin and albuminuria negatively correlate in patients with CKD.^90^ Adiponectin knockout mice exhibited albuminuria and podocyte foot process fusion. Administration of exogenous adiponectin was associated with reduced permeability to albumin by podocytes, seemingly caused by a reduction in oxidative stress.^90^

*Note*: This table summarizes evidence from both human studies and animal studies.

Abbreviations: CKD, chronic kidney disease; FSGS, focal segmental glomerulosclerosis; GFR, glomerular filtration rate; ICAM‐1, intercellular adhesion molecule‐1; IL‐6, interleukin 6; MCP‐1, monocyte chemo‐attractant protein 1; PAI, plasminogen‐activator inhibitor; ROS, reactive oxygen species; TGF‐β, transforming growth factor beta; TNF, tumor necrosis factor; UACR, urinary albumin/creatinine ratio; VCAM‐1, vascular cellular adhesion molecule‐1.

Moreover hypertriglyceridemia, a common abnormality noted in visceral obesity, is also implicated in the progression of kidney disease. CKD itself is also associated with dyslipidaemia, typically characterized by high levels of triglycerides, high levels of low‐density lipoprotein cholesterol, and low levels of high‐density lipoprotein cholesterol.^91^, ^92^ Abnormal lipid metabolism within this setting may be prompted by microalbuminuria, triggering a compensatory elevation of lipoprotein synthesis by the liver.^93^ Dyslipidaemia is also an important risk factor for CVD,^94^ and may contribute to renal injury by lipotoxicity, vascular injury, atherosclerosis, glomerulosclerosis, as well as inflammatory and oxidative stress mechanisms.^93^, ^95^

Furthermore, insulin resistance and associated hyperinsulinemia, important components of the metabolic syndrome, are pathophysiologically important in renal impairment, contributing to glomerular hyperfiltration, albuminuria, elevated vascular permeability, endothelial dysfunction, oxidative stress, and enhanced production of transforming growth factor beta and insulin like growth factor 1.^89^, ^96^, ^97^ Beyond the systemic effects of visceral fat, researchers were interested in the effects of perivascular adipose tissue and other locally acting fat depots which secrete adipokines, cytokines and specific angiogenic factors to the vascular wall.^98^ Renal sinus fat (RSF) is a perivascular fat depot located near renal arteries. Studies showed that increased RSF mass under exercise conditions aggravated microalbuminuria.^99^ In a study by Wagner et al., RSF was shown to induce kidney damage when acted on by hepatokine fetuin‐A (endogenous ligand for toll‐like receptor 4). There was no measurement of albuminuria to confirm the previously mentioned study. Some studies have shown that the genetic prediction of insulin resistance is not determined by BMI. This indicates that insulin resistance might also be relevant in lean people which can result in T2DM, CVD and CKD.^100^, ^101^

The association between obesity and renal tubular abnormalities has not been characterized to the same extent as obesity‐induced glomerular injury. However, in a study of human renal biopsy samples in patients with non‐diabetic obesity, the changes to tubular physiology included proximal tubular epithelial cell hypertrophy accompanied with greater proximal tubular volume.^49^ Similarly, histological analysis in a metabolic syndrome induced mouse model also showed signs of proximal tubular injury, thought to be instigated by increased levels of acid accumulation.^102^

## 4 OBESITY AND KIDNEY DISEASE: CLINICAL ASSOCIATIONS

The interaction between obesity and CKD has been delineated in numerous studies.^46^, ^103^, ^104^, ^105^ A high BMI is a recognized, graded and potent risk factor for renal impairment and ESKD within the general population.^46^, ^103^ A large body of evidence demonstrates that in contrast to individuals with a normal BMI, individuals with overweight and obesity are at a greater risk of CKD, seen also in patients with a “metabolically‐healthy” obese phenotype.^104^ Although the risk of ESKD is higher in people with metabolically healthy obesity compared to metabolically healthy people with normal weight, there is good evidence that people with metabolically healthy obesity have a lower risk compared to individuals with metabolically unhealthy obesity.^105^ The risk is similar for other adverse outcomes such as cardiovascular events and all‐cause mortality.^106^ Therefore, it is useful to adopt the concept of metabolically healthy obesity to encourage more people to lose weight and reduce the rate of adverse outcomes.^107^ To this end, a systematic review and meta‐analysis predicted that approximately 14% and 25% of males and females respectively develop CKD as a clinical consequence of overweight or obesity in industrialized countries.^108^

Obesity is a strong risk factor for kidney disease. A cohort study with greater than 320,000 patients showed a stepwise increase in the risk of ESKD with higher BMI. The relative risk of ESKD was 1.87, 3.57, 6.12, and 7.07 for overweight (BMI 25–29 kg/m^2^), class I obesity (BMI 30–34.9 kg/m^2^), class II obesity (BMI 35–39.9 kg/m^2^) and extreme obesity (BMI ≥40 kg/m^2^) respectively. The ESKD risk was consistently observed after adjustment for sex, race, and presence or absence of diabetes and hypertension.^103^ The mechanisms thought to underlie this involved those already mentioned in this review, including hypertension and diabetes which possess a causal relationship with CKD, as well as the harmful effects exerted by adiposity itself.^103^ Moreover, in a population‐based Swedish study of adults between the ages of 18 and 74 years, being overweight at the age of 20 years posed a three‐fold increased risk of CKD whilst obesity in males and clinically severe obesity in females at any age was linked to a three‐to‐four‐fold increase in risk, compared to lean adults. The risk was still three‐fold in analyses of patients without underlying diabetes and hypertension, again highlighting the causative association between adiposity and CKD onset.^107^

Silverwood et al.^109^ also outlined a greater risk of CKD with respect to early age of onset of overweight and obesity. In this study, based upon a cohort born in the year 1946 in England, Scotland and Wales, CKD at the ages of 60–64 years was twice as likely to occur if participants were overweight when assessed at the age timepoints of 26 and 36 years. The strength of the association declined significantly as the age of first‐onset obesity increased. In addition, the study suggested prevention of elevated BMI at any age is linked to a lower burden of CKD, but more so when obesity is prevented during early adulthood.^109^ Several other studies, with participants from diverse ethnic backgrounds, similarly report the association of a greater BMI conferring a larger risk of CKD onset and development.^110^, ^111^, ^112^, ^113^, ^114^

In addition to BMI, anthropometric measures such as waist circumference (WC) and waist/hip ratio (WHR) positively correlate as a risk determinant of CKD. This is perhaps because visceral adiposity poses a more potent metabolic risk than fat located elsewhere and is central to pathological sequelae caused by obesity such as insulin resistance and inflammation.^16^, ^115^ For example, Nerpin et al. showed a direct correlation between insulin sensitivity and subsequent eGFR level irrespective of BMI in patients with normal fasting glucose and normal glucose tolerance.^116^ Evans et al. found significant correlations between WHR and estimated GFR decline, as well as with an increase in urinary albumin‐creatinine ratio and uric acid levels.^117^ Central adipose distribution was also detrimental in lean persons in a cohort of 7676 non‐diabetic patients.^118^ In this study, participants with normal BMI and central adiposity (apple‐shaped) displayed diminished glomerular filtration, in a dose dependent manner; with increased visceral adiposity conferring increased CKD risk. This was similar in participants with overweight and obesity (pear‐shaped), though importantly, renal impairment was present irrespective of peripheral or central fat distribution in the context of obesity, in this study.^118^ In non‐diabetic patients with existing CKD, normal‐weight obesity (characterized by a normal BMI (<25 kg/m^2^) and a large proportion of body fat mass) was associated with the worst clinical outcome with regards to cardiovascular event composites and all‐cause mortality when compared to normal‐weight lean patients and patients with overweight and obesity. Highest levels of the inflammatory cytokine IL‐6 were also noted in patients with normal‐weight obesity.^119^

WC also positively associates with risk of all‐cause mortality in patients with CKD, and may prove an effective, relatively simple and time efficient tool to be used alongside BMI to assess mortality risk in population studies.^120^

Obesity is also documented to hasten renal deterioration in pathologies such as IgA nephropathy,^121^ and constitutes a greater level of risk for the developing nephrolithiasis.^122^ Non‐alcoholic fatty liver disease (NAFLD) is another important condition interlinking obesity and CKD.^120^ NAFLD is a condition characterized by the intrahepatic accumulation of fat in the form of triglycerides in people who take minimal or no alcohol. There is conflicting evidence regarding the link between NAFLD and CVD. Some studies shown NAFLD as a risk factor for CKD and CVD independent of the components of metabolic syndrome.^123^, ^124^ However, an exome‐wide association study identified genetic variants located at the 148Met allele in patatin‐like phospholipase domain‐containing protein 3, and Lysine at residue number 167 (167Lys) allele in transmembrane‐6 superfamily member 2 (TM6SF2) as strong factors for increased liver fat content, progression to cirrhosis, and protection from coronary artery disease.^125^ The discordance of the effects of these alleles is not well understood. In a 2017 study, Musso et al. studied the impact of polymorphism on lipoprotein metabolism in 60 individuals without obesity with NAFLD and a matched cohort. They concluded that the 167Lys allele in TM6SF2 might divert cholesterol from vessels into the liver inducing steatosis and cirrhosis.^126^

There are multiple putative mechanisms that interlink the pathogenesis of NAFLD and CKD including decreased adiponectin, activated protein kinase (AMPK) activity, and increased fibroblast growth factor 21 activity, increased fetuin‐A, mammalian target of rapamycin activity, and sodium–glucose cotransporter 2 (SGLT2) activity.^120^, ^127^ In general, an increased production of pro‐inflammatory cytokines and oxidative stress mechanisms promote the onset and progression of both NAFLD and CKD.^128^ NAFLD is also shown to be a strong and independent risk factor for CVD in people with advanced CKD.^129^ Despite the identification of multiple treatment targets, the only current proven management strategy for NAFLD is weight loss by lifestyle modification (diet and exercise)^130^ or bariatric surgery,^131^ which further strengthens the link between obesity and NAFLD.

## 5 WEIGHT MANAGEMENT CONSIDERATIONS IN KIDNEY DISEASE

Because surplus adiposity impacts negatively on renal health both in the general population and in those with existing CKD,^110^ targeting obesity is critical in the endeavor to reduce the national and global burden of the disease, and other ailments accelerated by excessive adiposity and metabolic syndrome. Fortunately, volitional weight management fosters good health outcomes in patients with obesity and reduces mortality,^132^ improves glycemic status and triglyceride levels, and better cardiometabolic outcomes are highlighted in the literature.^123^, ^133^ In consideration of renal health, targeted weight loss interventions improve outcomes such as microalbuminuria, proteinuria, hyperfiltration and can normalize GFR.^124^, ^134^, ^135^ Weight loss through caloric restriction and exercise is associated with decreases in body weight and fat proportion, as well as reduced inflammation and oxidative stress in patients with moderate‐to‐severe CKD.^136^ Bariatric surgery remains the most effective and enduring form of management of weight and comorbid conditions in patients with clinically severe obesity,^137^ and is associated with meaningful post‐surgical benefits in patients with CKD such as stabilized eGFR and significantly slower progression to ESKD.^134^, ^138^ Coleman et al. reported that bariatric surgery in pre‐dialysis CKD stages 3–5 and obesity stages II and III was associated with a reduction in mortality, irrespective of whether these patients developed ESKD.^139^

## 6 PHARMACOLOGICAL THERAPY TARGETS BOTH OBESITY AND CHRONIC KIDNEY DISEASE

In recent years, glucagon‐like peptide‐1 receptor agonists (GLP1 RA) and SGLT2 inhibitors emerged as effective glucose‐lowering agents with a good safety profile. Although they are licensed primarily to treat patients with T2DM, they proved to have other major positive outcomes including weight loss. SGLT2 inhibitors were proved to reduce the risk of ESKD, cardiovascular events, and mortality in patients with T2DM and CKD.^140^ The effects of SGLT2 inhibitors were proved to go beyond the glucose‐lowering effect by showing similar outcomes in people without T2DM but with CKD and proteinuria.^141^ Although SGLT2 inhibitors induce weight loss, they are not currently licensed for people with CKD and obesity with no significant proteinuria and/or T2DM. However, they work on different aspects of the metabolic syndrome and can indirectly benefit patients with obesity. The primary outcomes of several studies showed that GLP‐1 RAs reduce major cardiovascular events and all‐cause mortality in patients with T2DM. Trials also showed that they may have a role in slowing the progression of CKD in patients with T2DM as per secondary outcome data.^142^, ^143^, ^144^, ^145^ In an animal study, liraglutide, a GLP‐1 RA, restored renal metabolism by inhibiting renal lipid accumulation and rescued renal mitochondria function after inducing obesity in rats using a high fat diet.^146^ These data are encouraging to consider GLP‐1 RAs in people with obesity and CKD in future clinical trials.

## 7 OBESITY AND DIALYSIS

In patients receiving maintenance hemodialysis [maintenance hemodialysis (MHD)], abdominal adiposity was linked to inflammatory processes and protein energy wasting, and is highlighted as a risk factor for mortality.^147^ In a Korean study of 18,699 participants all receiving MHD; whilst a higher BMI associated negatively with mortality; increased WC in fact conferred a higher risk of mortality.^148^ Moreover, Wang et al.^149^ observed that lean body mass, which accounts for the proportion of adipose‐free body mass, associated negatively with mortality risk. The proportion of lean body mass can remain greatly unaccounted for in measures of BMI as a person with a large proportion of skeletal muscle can be classified in the overweight or obese categories of BMI.^117^ With this in mind, it is important to reference that sarcopenia, which describes a loss in skeletal muscle mass and strength, is a common comorbidity within the elderly MHD population and is associated with considerable levels of frailty, protein energy wasting and disability. Sarcopenic obesity can also occur whereby sarcopenia can co‐exist with obesity, and this is associated with a lower quality of life, greater risk of frailty, mortality and inflammation in ESKD and in patients in receipt of MHD.^150^, ^151^

With regards to patients receiving peritoneal dialysis (PD), previous reports have shown that obesity is associated with worse treatment outcomes, in relation to treatment survival rate, frailty and risk of mortality.^152^, ^153^ A significantly greater risk of peritonitis has also been noted in some, though not all studies.^154^, ^155^ Due to raised intra‐abdominal pressure, a higher incidence of peritoneal leaks is also observed in the population of patients receiving PD who have obesity.^156^ Severe obesity often may pose a contraindication to being treated with PD.^157^, ^158^ Obi et al.^159^ demonstrated that patients with a higher BMI were transferred more quickly to receive hemodialysis, were at a greater risk of peritonitis‐related complications and had quicker deterioration in residual renal function. However, in patients with severe obesity (BMI >35 kg/m^2^), the survival was analogous to matched MHD patients.^159^

## 8 OBESITY AND KIDNEY TRANSPLANTATION

Obesity is common both amongst recipients of and donors for kidney transplantation^156^ and it contraindicates transplantation as a feasible treatment option in many patients.^160^ Nevertheless, renal transplantation is the preferred treatment for ESKD and is associated with better longevity and quality of life in contrast to dialysis.^161^ Indeed, studies have observed a several‐fold greater survival advantage with transplantation in ESKD patients with obesity, more so than those who remained on the waiting list and receiving dialysis.^162^, ^163^ Nevertheless, obesity is classified as a risk factor, because of a range of surgical difficulties namely prolonged operative time, delayed wound healing, higher risk of venous‐thromboembolism, peripheral nerve injury and incidence of cardiac events.^164^, ^165^, ^166^ Many studies have shown that obesity at the time of transplantation correlates independently with delayed wound healing, wound complication, graft loss and greater duration of hospitalization; along with a larger burden of readmissions 1‐year post‐transplant, increased CVD, diabetes mellitus risk and premature patient death in studies across the literature.^161^, ^167^, ^168^, ^169^ In contrast, there are studies that have observed similar outcomes post‐kidney transplant,^163^, ^170^, ^171^ in patients with or without obesity; suggesting that renal transplantation may be conducted safely within this subpopulation. For example, an analysis from the United Kingdom transplant registry noted that the survival advantage of kidney transplantation in ESKD was greater across all BMI bands.^163^ Marks et al. observed comparable results with regards to patient survival and graft survival in patients with clinically severe obesity in comparison to those without obesity 3 years post‐transplant, although the patients with clinically severe obesity displayed longer in‐hospital stays, and higher rates of readmission and major wound complications.^172^

Whether weight management should be advocated in patients with obesity prior to receiving renal transplantation requires further research; some studies have reported that substantial loss in body weight before transplantation is seen to pose either no observable benefit,^173^ or might in fact increase the risk of adverse events such as lengthened hospitalization and graft loss.^174^ Greater mortality risk is also detected in some studies, potentially as reductions in BMI may be associated with processes such as protein malnutrition, muscle mass loss and systemic inflammation.^174^, ^175^ Therefore, volitional weight loss efforts may require robust monitoring, with consideration of unique patient adjustments in those awaiting renal transplantation.^173^ Bariatric surgery however, prior to renal transplantation, is observed to be a safe, feasible and beneficial procedure,^176^, ^177^ which improves surgical access and kidney transplantation in patients with clinically severe obesity.

## 9 CLINICAL IMPLICATIONS AND FUTURE RESEARCH

Obesity is a tangible risk factor for the development and progression of CKD.^105^ The prevalence of kidney disease amongst patients with overweight or obesity varies amongst studies, although in a systematic review and meta‐analysis it was reported that approximately 24% and 34% of kidney disease cases amongst American males and females, respectively, were related to overweight and obese.^108^

The rising prevalence of CKD is paralleled by an increase in the aging population, as well as the number of individuals with obesity, type 2 diabetes and hypertension; resulting in a greater population of individuals who are potentially susceptible to CKD.^178^ With a considerable escalation in the incidence of CKD and associated risk of ESKD there will be a greater number of people requiring RRT^179^ over the coming decades, and this should influence changes at the population level aimed at reducing the initiation and progression of CKD. Modifiable risk factors including overweight or obesity should be targeted for prevention implementation strategies such as healthy eating, physical activity and improved energy balance to counteract the onset and worsening of kidney disease. In a systematic review of trials studying lifestyle and behavioral modifications in adults with CKD,^180^ approximately 70% of studies demonstrated significant improvement in measured physiological metrics such as GFR, blood pressure, albumin excretion and body composition with the implementation of lifestyle change. Commonly utilized interventions and behavioral techniques included patient education, enablement, social support and feedback and structure, whilst education was the basis for the most promising intervention in terms of achieving physiological improvement.^180^ The need for larger population and epidemiological‐based studies to assess the efficacy of lifestyle‐based approaches in managing CKD, and the subsequent scope of implementing standardized approaches to lifestyle modification, is recognized.

The postulated mechanisms by which overweight and obesity may contribute toward the development and progression of CKD require further research, although there is an appreciation that adiposity both directly and indirectly affects renal function. The metabolic complications of obesity exert pathophysiological changes in renal performance, including glomerular dysfunction.^108^ Excessive adipose tissue in individuals with obesity may be associated with an adverse hormonal and cytokine profile, ectopic lipid accumulation and altered renal hemodynamics which result in nephrotoxicity and latent changes such as ORG, causing proteinuria and a spectrum of wider renal dysfunction.^44^ However, a significant proportion of patients with overweight, obesity and/or components of the metabolic syndrome will not develop CKD,^42^ and the reasons for this differentiation of risk are unclear. Future insight should build on genetic, epigenetic and phenotypic understanding of why certain individuals may be more inclined to develop renal impairment secondary to these metabolic conditions, and to explore other potential mechanisms of kidney injury.

In patients with CKD and obesity, WC possesses a greater correlation with adverse renal outcomes and mortality compared to overall BMI. This may be linked, at least in part, to the higher risk associated with visceral fat in terms of metabolic outcomes and future cardiovascular events, in comparison to peripheral or gluteo‐femoral adiposity.^181^ Ongoing clinical studies and routine patient care may benefit from the incorporation of WC measurements into daily practice.^117^

The main strategies utilized to manage obesity in patients with CKD are lifestyle modification and bariatric surgery. Bariatric surgery remains effective in treating clinically severe obesity, type 2 diabetes and hypertension, although its therapeutic effects extend beyond weight reduction. Indeed, studies have shown that bariatric surgery may be associated with decreased risk of CKD onset^182^ and progression to ESKD^183^ in at‐risk individuals, and the surgical risk appears only slightly greater in comparison to the general bariatric surgery population.^183^ Benefits of bariatric surgery before renal transplantation are also recorded in the literature.^177^ However, assessment is required to carefully and judiciously weigh up the benefits of bariatric surgery in patients with clinically severe obesity and CKD, compared to potential risks and thus, rigorous patient selection is necessary to optimize outcomes.^184^ There is also a need to focus research on the benefits of bariatric surgery in lower risk groups who are currently not eligible for it.

Furthermore, future research should explore the benefits of pharmaco‐therapeutic means to treat obesity, either alone, or in synergy with surgery. SGLT2 inhibitors and GLP‐RAs may be beneficial in this cohort of patients due to their multi‐faceted actions in improving several outcomes such as weight loss, glycemic control, progression of CKD, cardiovascular events and all‐cause mortality.^140^ Recent contemporary advances in the treatment of obesity and diabetes has also guided the development of co‐agonistic approaches to drug therapy such as GLP‐1/glucagon, glucose‐dependent insulinotropic polypeptide (GIP)–GLP1, amylin–calcitonin dual agonists, and GIP–GLP1–glucagon tri‐agonists.^185^ Ongoing studies should determine the efficacious properties of these drugs in a population of patients with overweight/obesity and CKD, so as to explore the potential of narrowing the therapeutic gap between pharmacotherapy and surgical treatment of obesity in this cohort.

## AUTHOR CONTRIBUTIONS

Saira Nawaz wrote the first draft of the manuscript. Saif Al‐Chalabi and Saira Nawaz wrote the final version of the manuscript. Rajkumar Chinnadurai, Philip Evans, Philip A. Kalra, Akheel A. Syed, and Smeeta Sinha revised the manuscript and made corrections. All authors approved the final manuscript.

## CONFLICT OF INTEREST

Smeeta Sinha reports receiving research funding from Amgen, Ethicon, and AstraZeneca and receiving honoraria from Astra Zeneca, Napp Pharmaceuticals, Vifor Pharma, Novartis, Bayer, and Sanofi‐Genzyme. Philip A. Kalra reports grants from Vifor and receiving Honoria from Astra Zeneca, Astellas, Pharmacosmos, Boehringer Ingelheim, Napp, and Vifor. The other authors have nothing to disclose.
